# The long non-coding RNA, urothelial carcinoma associated 1, promotes cell growth, invasion, migration, and chemo-resistance in glioma through Wnt/β-catenin signaling pathway

**DOI:** 10.18632/aging.102317

**Published:** 2019-10-08

**Authors:** Beilin Zhang, Shaokuan Fang, Yingying Cheng, Chunkui Zhou, Fang Deng

**Affiliations:** 1 Department of Neurology, The First Teaching Hospital of Jilin University, Changchun, Jilin 130021, P.R. China

**Keywords:** urothelial carcinoma associated 1, glioma, cell proliferation, apoptosis, Wnt/β-catenin

## Abstract

The long non-coding RNA, urothelial carcinoma associated 1 (UCA1) has been demonstrated to play important roles in various types of cancers. This study investigated the functional role of UCA1 in glioma and explored the underlying molecular mechanisms. UCA1 was found to be highly up-regulated in glioma cells, and knock-down of UCA1 inhibited cell growth, invasion and migration, and also induced apoptosis in glioma cells. On the other hand, overexpression of UCA1 promoted cell proliferation, cell invasion and migration in glioma cells. Knock-down of UCA1 suppressed the activity of Wnt/β-catenin signaling, and treatment with lithium chloride restored the inhibitory effect of UCA1 knock-down on cell invasion and migration. More importantly, the aberrant expression of UCA1 was associated with chemo-resistance to cisplatin and temozolomide in glioma cells via interacting with Wnt/β-catenin signaling. *In vivo* studies showed that overexpression of UCA1 promoted the *in vivo* tumor growth of U87 cells in the nude mice. Clinically, UCA1 was found to be up-regulated in glioma tissues and higher expression level of UCA1 was correlated with poor survival in patients with glioma. Taken together, our results showed that UCA1 had a functional role in the regulation of glioma cell growth, invasion and migration, and chemo-resistance possibly via Wnt/β-catenin signaling pathway.

## INTRODUCTION

Glioma is one of the most deadly cancers of the central nervous system, which accounts for about 80% of primary malignant brain tumors [1]. Glioblastoma is the most common type of glioma and is associated with very poor survival in patients [2]. Currently, the treatment for glioma is surgery in combination with chemotherapy [3, 4]. Unfortunately, there is a substantial number of recurrence after surgical treatment [5]. The pathogenesis of glioma, particularly its underlying molecular mechanism, is largely unknown. Studies have found that gene mutation plays important roles in the pathogenesis of glioma [6]. Recently, epigenetic regulation and non-coding RNAs are also found to contribute to glioma development [7, 8]. However, the detailed mechanisms underlying non-coding RNAs in the pathogenesis of glioma are not fully addressed. Understanding the role of non-coding RNAs in glioma will be helpful for us to identify novel therapy for this malignancy.

The long non-coding RNA (lncRNA) has drawn great attention due to the diverse roles in the cellular processes such as gene translation, metabolism, cell growth and proliferation [9]. The lncRNA consists of more than 200 nucleotides and can not be translated into protein [9]. According to previous studies, lncRNAs were found to exert the functions through different mechanisms, particularly in the regulation of tumor development. The lncRNAs are found to act as a sponge for endogenous microRNAs (miRNAs). For example, the lncRNA, nuclear paraspeckle assembly transcript 1 interacted with miR-101 to modulate breast cancer growth by targeting EZH2 [10]; the lncRNA, SPRY4 intronic transcript 1 sponges miR-101-3p to promote proliferation and metastasis of bladder cancer cells through up-regulating EZH2 [11]. In addition, the lncRNA, X-inactive specific transcript promotes cell growth and invasion through regulating miR-497/metastasis associated in colon cancer 1 axis in gastric cancer [12]. The lncRNAs are also found to regulate gene expressions via the epigenetic mechanisms. The lncRNA maternally expressed 3 contributes to the epigenetic regulation of epithelial-mesenchymal transition in lung cancer cells [13]; the lncRNA, H19 confers chemoresistance in estrogen alpha-positive breast cancer through epigenetic silencing of the pro-apoptotic gene BCL2 interacting killer [14]. The lncRNA, urothelial carcinoma associated 1 (UCA1) has been studied in a broad range of cancer types such as breast cancer, melanoma, gastric cancer, colorectal cancer, bladder cancer and others, and various mechanism underlying the regulatory role of UCA1 in cancer development have been proposed [15–18].

Wnt/β-catenin signaling pathway plays essential roles in human malignancies including glioma [19]. Disruption of Wnt/β-catenin signaling has been shown to inhibit the tumorigenesis of glioma [20]. Recently, studies found that UCA1 was effective to enhance the activities of Wnt/β-catenin in several types of cancers including melanoma [21], breast cancer [17], osteosarcoma [22] and oral squamous cell carcinoma [23]. However, whether UCA1 interacts with Wnt/β-catenin signaling remains elusive.

In the present study, we identified the up-regulation of UCA1 in glioma cell lines, and further functional study showed that UCA1 knock-down inhibited cell growth, cell invasion and migration, and also induced cell apoptosis. Overexpression of UCA1 promoted cell proliferation, cell invasion and migration in glioma cells, and promoted *in vivo* tumor growth of glioma cells. UCA1 dysregulation was found to be associated with the chemosensitivity in glioma cells. More importantly, we found that higher expression of UCA1 in glioma tissues are associated with poor survival of glioma patients.

## RESULTS

### Up-regulation of UCA1 in glioma cells

**Figure 1:**
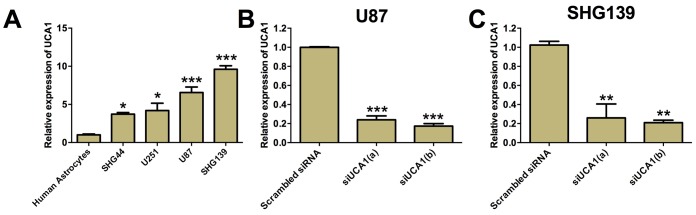
**UCA1 was up-regulated in glioma cell lines.** (**A**) The expression of UCA1 in human astrocytes and glioma cell lines was determined by qRT-PCR. UCA1 was up-regulated in glioma cell lines (SGH44, U251, U87 and SHG139). The expression of UCA1 in (**B**) U87 cells and (**C**) SHG139 cells after UCA1 siRNAs (siUCA1(a) and siUCA1(b)) or scrambled siRNA transfection was determined by qRT-PCR. All the experiments were performed in triplicates. Significant differences compared to the control group were expressed as *P<0.05, **P<0.01 and ***P<0.001.

UCA1 was found to play important roles in various types of cancers. Two different transcripts of UCA1 (~1.4 kb or ~2.3 kb) have been reported previously [24, 25], and in the present study, we determined expression of UCA1 (~1.4 kb) based on the previous study [26]. We examined the expression of UCA1 in glioma cell lines including SHG44, U251, U87 and SHG139 cells as well as human astrocytes by using qRT-PCR. The expression of UCA1 in glioma cells were normalized to that of human astrocytes. It was found that the expression of UCA1 in SHG44, U251, U87 and SHG139 cells were significantly higher than that in human astrocytes (Figure 1A, P<0.05). As UCA1 was up-regulated in the glioma cell lines, we chose the glioma cell lines, U87 and SHG139 that have the highest expression of UCA1 for the loss-of-function study. Two UCA1 siRNAs were designed to knock-down the expression of UCA1 in U87 and SHG139 cells. As shown in Figure 1B and Figure 1C, the UCA1 siRNAs (UCA1(a) and UCA1(b)) transfection significantly suppressed the expression of UCA1 in U87 and SHG139 cells as compared to cells transfected with scrambled siRNA (Figure 1B and 1C, P<0.05).

### Knock-down of UCA1 inhibited cell proliferation and induced apoptosis in glioma cells

**Figure 2:**
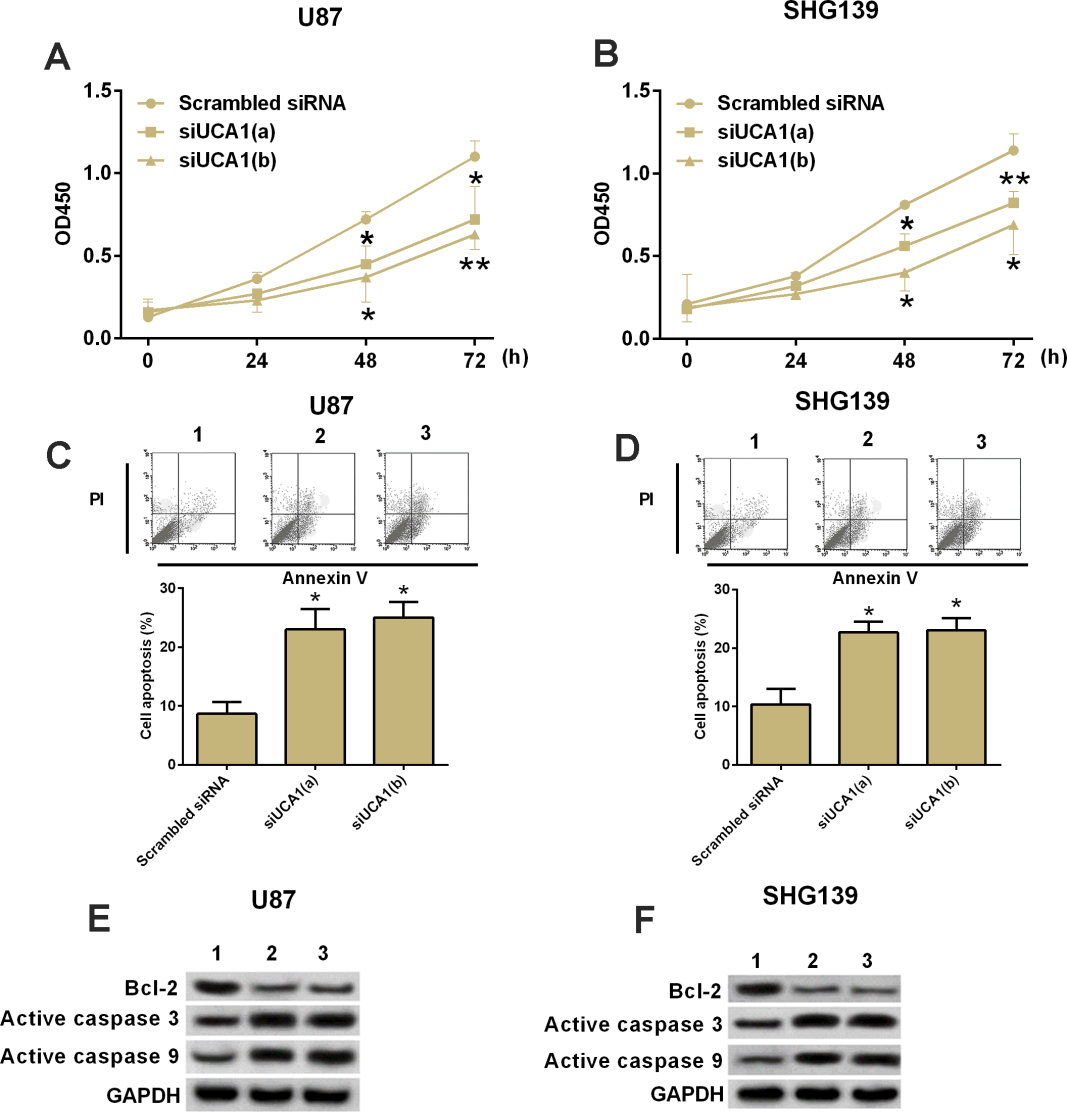
**Knock-down of UCA1 inhibited cell growth and increased apoptotic rate in glioma cells.** (**A** and **B**) CKK-8 assay. The cell growth in (**A**) U87 and (**B**) SHG139 cells at 0, 24, 48 and 72 h after UCA1 siRNAs (siUCA1(a) and siUCA1(b)) or scrambled siRNA transfection. (**C** and **D**) Flow cytometry. The cell apoptotic rate in (**C**) U87 and (**D**) SHG139 cells after UCA1 siRNAs (siUCA1(a) and siUCA1(b)) or scrambled siRNA transfection. (**E** and **F**): Western blotting. The protein expression of Bcl-2, active caspase 3, and active caspase 9 in (**E**) U87 and (**F**) SHG139 cells after UCA1 siRNAs (siUCA1(a) and siUCA1(b)) or scrambled siRNA transfection. 1, scrambled siRNA; 2, siUCA1(a); 3, siUCA1(b). All the experiments were performed in triplicates. Significant differences compared to the control group were expressed as *P<0.05 and **P<0.01.

CCK-8 assay was performed to determine the cell proliferation in U87 and SHG139 cells after UCA1 siRNAs transfection. The results showed that glioma cells transfected with UCA1 siRNAs had significantly lower growth rate of glioma cells at 48 and 72 h post UCA1 siRNAs transfection than cells transfected with scrambled siRNA (Figure 2A and 2B, P<0.05). Furthermore, we performed flow cytometry experiment to examine the cell apoptotic rate in U87 and SHG139 cells after UCA1 siRNAs transfection. The results showed that UCA1 siRNAs transfection significantly increased the cell apoptotic rate in U87 and SHG139 cells as compared to scrambled siRNA transfection (Figure 2C and 2D, P<0.05). To understand the change of protein biomarkers related to the knock-down of UCA1 on cell apoptosis in U87 and SHG139 cells, western blotting was performed, and the results showed that knock-down of UCA1 by UCA1 siRNAs transfection in U87 and SHG139 cells significantly increased the protein expression of active caspase 3 and active caspase 9 and decreased the protein expression of Bcl-2 when compared to cells transfected with scramble siRNA (Figure 2E and 2F, P<0.05).

### Knock-down of UCA1 inhibited cell invasion and cell migration in glioma cells

**Figure 3:**
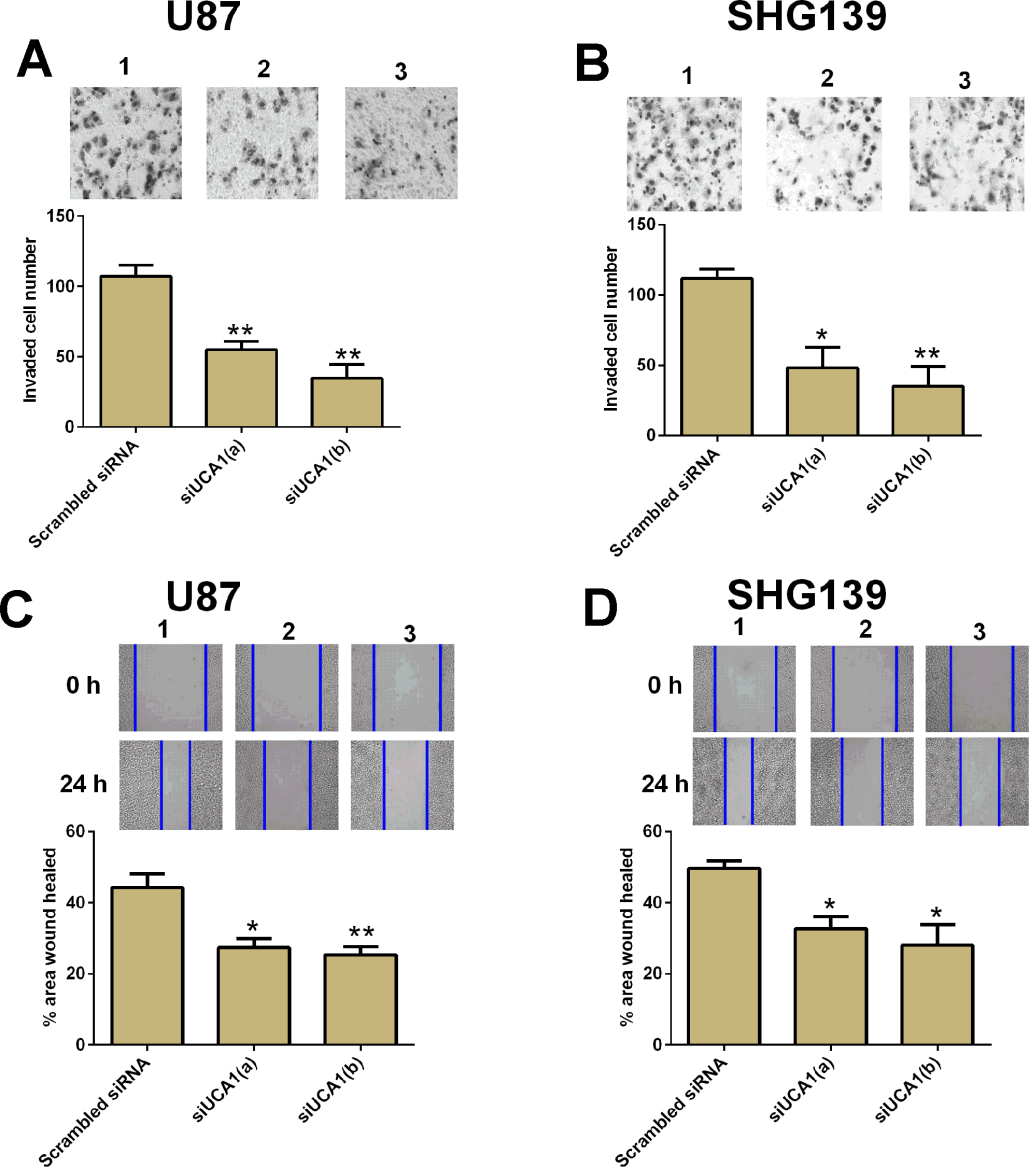
**Knock-down of UCA1 inhibited cell invasion and migration in glioma cells.** The cell invasive potential in (**A**) U87 and (**B**) SHG139 cells after UCA1 siRNAs (siUCA1(a) and siUCA1(b)) or scrambled siRNA transfection was determined by cell invasion assay. The cell migratory potential in (**C**) U87 and (**D**) SHG139 cells after UCA1 siRNAs (siUCA1(a) and siUCA1(b)) or scrambled siRNA transfection were assessed by cell migration assay. 1, scrambled siRNA; 2, siUCA1(a); 3, siUCA1(b). All the experiments were performed in triplicates. Significant differences compared to the control group were expressed as *P<0.05 and **P<0.01.

Cell invasion and cell migration contributed to tumor metastasis. In this study, we performed cell invasion and cell migration assay to examine the role of UCA1 in cell invasion and migration in U87 and SHG139 cells. The U87 and SHG139 cells transfected with UCA1 siRNAs showed a decrease in the invaded cell number compared to cells transfected with scrambled siRNA (Figure 3A and 3B, P<0.05). The wound healing assay results showed that UCA1 siRNAs transfection significantly suppressed the cell migratory ability in U87 and SHG139 cells when compared to scrambled siRNA transfection (Figure 3C and 3D, P<0.05).

### UCA1 overexpression promoted cell proliferation, cell invasion and migration in glioma cells

**Figure 4:**
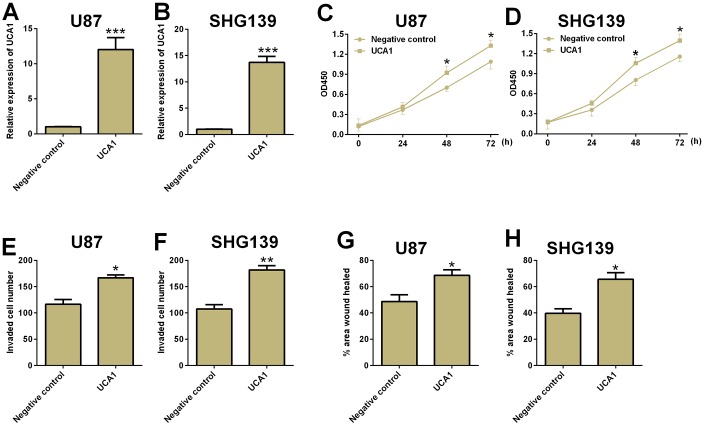
**Overexpression of UCA1 promoted cell proliferation, cell invasion and migration in glioma cells.** The relative expression of UCA1 in (**A**) U87 and (**B**) SHG139 cells after pcDNA3.1-UCA1 (UCA1) or pcDNA3.1 (negative control) transfection was determined by qRT-PCR assay. The cell proliferative potential in (**C**) U87 and (**D**) SHG139 cells after pcDNA3.1-UCA1 (UCA1) or pcDNA3.1 (negative control) transfection was determined by CCK-8 assay. The cell invasive potential in (**E**) U87 and (**F**) SHG139 cells after pcDNA3.1-UCA1 (UCA1) or pcDNA3.1 (negative control) transfection was determined by cell invasion assay. The cell migratory potential in (**G**) U87 and (**H**) SHG139 cells after pcDNA3.1-UCA1 (UCA1) or pcDNA3.1 (negative control) transfection by wound healing assay. All the experiments were performed in triplicates. Significant differences compared to the control group were expressed as *P<0.05, **P<0.01 and ***P<0.001.

The effects of UCA1 overexpression on glioma cell proliferation, cell invasion and migration were determined by CCK-8, cell invasion and wound healing assays, respectively. As shown in Figure 4, overexpression of UCA1 significantly increased cell proliferation, cell invasion and cell migration in both U87 and SHG193 cells (Figure 4).

### UCA1 regulated cell invasion and migration in glioma cells via Wnt/β-catenin signaling

**Figure 5:**
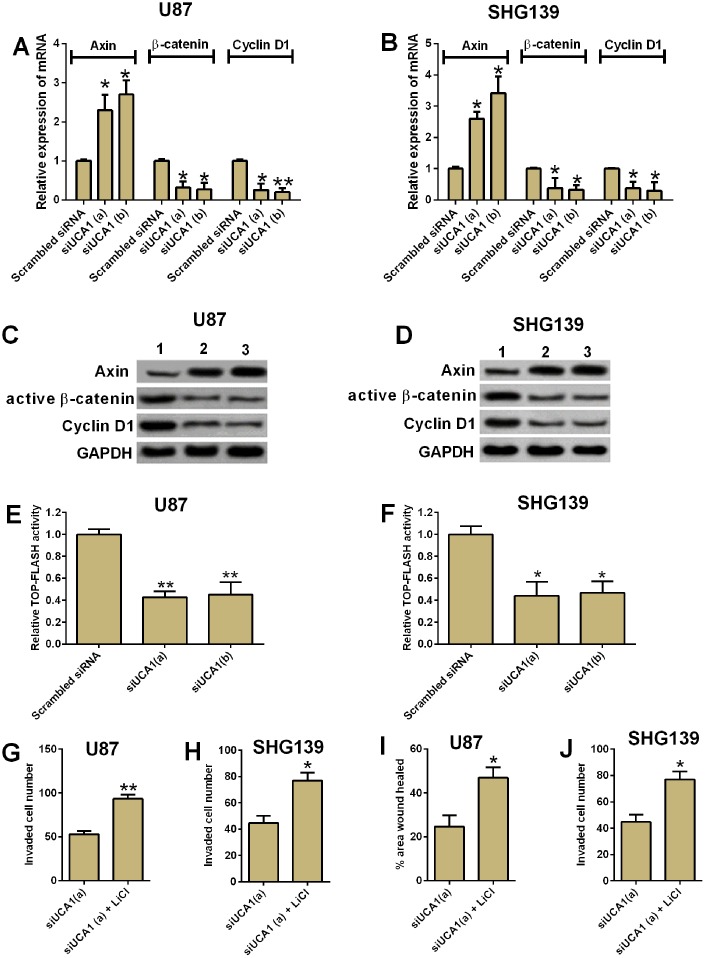
**UCA1 regulated glioma cell invasion and migration via Wnt/β-cateinin signaling pathway.** The mRNA expression of axin, β-cateinin and cyclin D1 in (**A**) U87 and (**B**) SHG139 cells after UCA1 siRNAs (siUCA1(a) and siUCA1(b)) or scrambled siRNA transfection were determined by qRT-PCR. The protein expression levels of Axin, active β-cateinin and cyclin D1 in (**C**) U87 and (**D**) SHG139 cells after UCA1 siRNAs (siUCA1(a) and siUCA1(b)) or scrambled siRNA transfection were determined by western blotting assay. The TOP-FLASH activity of (**E**) U87 and (**F**) SHG139 cells after UCA1 siRNAs (siUCA1(a) and siUCA1(b)) or scrambled siRNA transfection were determined by TOP-FLASH assay. The cell invasive potential in (**G**) U87 and (**H**) SHG139 cells after treatment with siUCA1(a) or siUCA1(a) + LiCl was determined by cell invasion assay. The cell migratory potential in (**I**) U87 and (**J**) SHG139 cells after treatment with siUCA1(a) or siUCA1(a) + LiCl was determined by cell migration assay. 1, scrambled siRNA; 2, siUCA1(a); 3, siUCA1(b). All the experiments were performed in triplicates. Significant differences compared to the control group were expressed as *P<0.05 and **P<0.01.

The Wnt/β-catenin is an important signaling pathway for tumor development in various types of cancers. We performed both qRT-PCR and western blotting assay to examine the mRNA and protein expression of several important mediators in the Wnt/β-catenin signaling pathway. The knock-down of UCA1 significantly decreased the mRNA and protein expression of active β-catenin, cyclin D1, and increased the mRNA and protein expression of axin (Figure 5A–5D, P<0.05). In addition, knock-down of UCA1 reduced the TOP-FLASH activity in U87 and SNH139 cells. In order to further confirm that the role of UCA1 in glioma cell invasion and migration involved in Wnt/β-catenin signaling, we treated the U87 and SHG139 cells with both UCA1 siRNA and LiCl (GSK3β inhibitor, which activates the Wnt/β-catenin signaling). Treatment with LiCl significantly increased the protein levels of active β-catenin and the TOP-FLASH activity (see Supplementary Figure 1). The cell invasion and cell migration assays showed that co-treatment with UCA1 siRNA and LiCl in U87 and SHG139 cells significant restored the inhibitory effect of UCA1 siRNA transfection on cell invasion and cell migration (Figure 5E–5H, P<0.05).

### UCA1 overexpression promoted chemo-resistance in glioma cells

**Figure 6:**
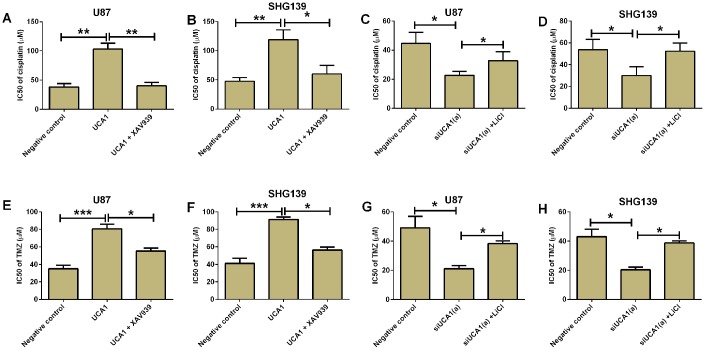
**UCA1 promoted chemo-resistance in glioma cells.** The IC50 of cisplatin for (**A**) U87 and (**B**) SHG139 cell lines in negative control (pcDNA3.1), UCA1 (pcDNA3.1-UCA1), and UCA1 (pcDNA3.1-UCA1) + XAV939 groups was determined by CCK-8 assay. The IC50 of cisplatin for (**C**) U87 and (**D**) SHG139 cell lines in negative control (scrambled siRNA), siUCA1(a), siUCA1(a) + LiCl groups was determined by CCK-8 assay. The IC50 of TMZ for (**E**) U87 and (**F**) SHG139 cell lines in negative control (pcDNA3.1), UCA1 (pcDNA3.1-UCA1), and UCA1 (pcDNA3.1-UCA1) + XAV939 groups was determined by CCK-8 assay. The IC50 of TMZ for (**G**) U87 and (**H**) SHG139 cell lines in negative control (scrambled siRNA), siUCA1(a), siUCA1(a) + LiCl groups was determined by CCK-8 assay. All the experiments were performed in triplicates. Significant differences compared to the control group were expressed as *P<0.05, **P<0.01 and ***P<0.01.

UCA1 was found to promote chemo-resistance in various types of cancers. The IC50 of cisplatin on glioma cells was evaluated using CCK-8 assay. The results showed that overexpression of UCA1 in U87 and SHG139 cells significantly increased the IC50 of cisplatin on these cells when compared to negative control group. Treatment with XAV939 (Wnt/β-catenin signaling inhibitor) in UCA1-overexpressing U87 and SHG139 cells significantly decreased the IC50 of cisplatin (Figure 6A and 6B, P<0.05). More importantly, the knock-down of UCA1 in U87 and SH139 cells also decreased the IC50 of cisplatin as compared to cells transfected with scramble siRNA, and LiCl treatment restored the inhibitory effect of UCA1 siRNA transfection on glioma cell chemo-resistance (Figure 6C and 6D). Consistently, overexpression of UCA1 in U87 and SHG139 cells significantly increased the IC50 of temozolomide (TMZ), which was attenuated by XAV939 treatment (Figure 6E and 6F, P<0.05); on the other hand, UCA1 knock-down decreased the IC50 of TMZ in U87 and SHG139 cells, which was partially restored by LiCl treatment (Figure 6G and 6H, P<0.05).

### UCA1 overexpression promoted in vivo tumor growth in nude mice

**Figure 7:**
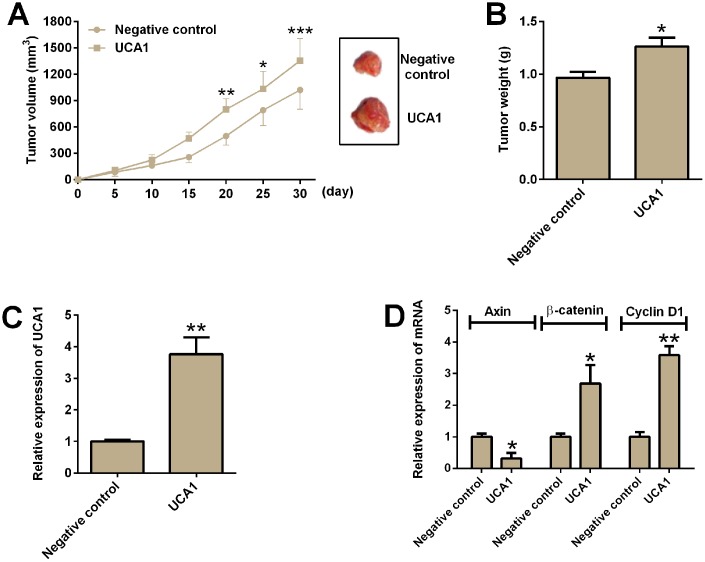
**UCA1 promoted *in vivo* tumor growth in the nude mice.** (**A**) The tumor growth of U87 cells with UCA1 overexpression or control U87 cells were assessed in the nude mice. Right panel showed the dissected tumors from the nude mice. (**B**) Tumor weight was determined in the isolated tumors from the nude mice. (**C**) The relative expression of UCA1 was determined by qRT-PCR in the isolated tumor tissues from control group and UCA1-overexpresssing group. (**D**) The relative mRNA expression of axin, β-catenin and cyclin D1 was determined by qRT-PCR in the isolated tumor tissues from control group and UCA1-overexpressing group. N = 5; significant differences compared to the control group were expressed as *P<0.05, **P<0.01 and ***P<0.001.

The effects of UCA1 overexpression on *in vivo* tumor growth were assess in a xenograft nude mice model. U87 cells with UCA1 overexpression or control U87 cells with pcDNA3.1 transfection were inoculated into the nude mice. The tumor volume was measured every 5 days for up to 30 days, and U87 cells with UCA1 overexpression showed significantly larger volume of tumor in the nude mice than that in the negative control group (Figure 7A). The tumor weight of the isolated tumors in the UCA1 group was significantly increased when compared to negative control group (Figure 7B). In addition, qRT-PCR analysis of relevant gene expression levels in the isolated tumor tissues showed that the expression of UCA1, β-catenin and cyclin D1 was up-regulated, while the expression of axin was down-regulated from the UCA1 group (Figure 7C and 7D).

### UCA1 was up-regulated in glioma tissues and correlated with poor clinical outcomes in patients with glioma

**Figure 8:**
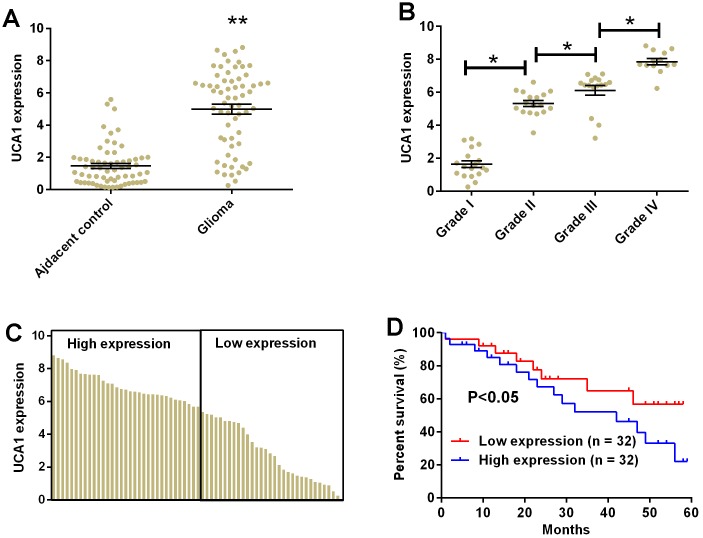
**UCA1 up-regulated in glioma tissues and correlated with poor clinical outcomes in patients with glioma.** (**A**) The expression of UCA1 in glioma tissues (n = 64) and adjacent normal brain tissues (n = 64) determined by qRT-PCR. (**B**) The expression of UCA1 in glioma tissues from patients with tumor grade I (n = 18), grade II (n = 17), grade III (n = 16), and grade IV (n = 13) was measured by qRT-PCR. (**C**) The expression of UCA1 in every glioma tissue was determined by qRT-PCR. (**D**) The overall survival of glioma patients with low or high expression of UCA1 in glioma tissues were assessed by Kaplan-Meier survival analysis. Significant differences compared to control group were expressed as *P<0.05 and **P<0.01.

The clinical samples from patients with glioma were collected for the analysis of UCA1 by qRT-PCR. The expression of UCA1 in glioma tissues were significantly higher than that in adjacent normal brain tissues (Figure 8A, P<0.05). We further compared the expression of UCA1 in different groups of patients based on the tumor grade and found that the expression of UCA1 was positively correlated with the tumor grade, i.e. the more advance tumor grade, and higher expression level of UCA1 in the glioma tissues (Figure 8B, P<0.05). The levels of UCA1 in glioma tissues were divided into low and high expression based on the median expression level of UCA1 (Figure 8C). The Kaplan-Meier curve analysis was performed to compare the overall survival in patients with low expression of UCA1 (n =32) and high expression of UCA1 (n =32). Patients with high expression level of UCA1 in glioma tissues had significantly lower 5-year survival rate than those with low expression level of UCA1 (P<0.05, hazard ratio = 2.156, 95% confidence interval = 1.341-5.014, P<0.05; Figure 8D).

## DISCUSSION

The role of UCA1 in glioma was investigated in the present study. Our results showed that UCA1 was up-regulated in glioma cells, and knock-down of UCA1 inhibited glioma cell growth, cell invasion and migration. It was also showed that knock-down of UCA1 induced cell apoptosis and inhibited Wnt/β-catenin signaling activity. On the other hand, overexpression of UCA1 promoted cell proliferation, cell invasion and migration of glioma cells. In addition, UCA1 promotes chemo-resistance via Wnt/β-catenin signaling. *In vivo* studies showed that overexpression of UCA1 promoted the *in vivo* tumor growth of U87 cells in the nude mice. In the clinical aspects, we found that UCA1 was up-regulated in glioma tissues and higher expression of UCA1 was positively correlated with the advanced tumor grade and predicted poor survival of glioma patients.

UCA1 was first identified in bladder cancer and functions to regulate embryonic development and promote bladder cancer invasion and migration [25]. After that, UCA1 was also found to be dysregulated in other types of cancers, and UCA1 had different mechanisms in regulating cancer progression. Previous studies showed that UCA1 shared a common role as an endogenous sponge for miRNAs. In melanoma, UCA1-miR-507-forkhead box protein M1 axis is involved in cell proliferation, invasion and G0/G1 cell cycle arrest [27]; in breast cancer, UCA1 functions as a competing endogenous RNA to suppress epithelial ovarian cancer metastasis [28]; UCA1 was also found to exert its oncogenic function in non-small cell lung cancer by targeting miR-193a-3p [29]. In addition, UCA1 was found to be epigenetically regulated by special AT-rich sequence binding protein 1 to regulate breast cancer cell growth and survival [30]. The role of UCA1 in cell apoptosis was also found in several types of cancers such as bladder cancer, breast cancer, gastric cancer and others [17, 31–33]. Consistently, our results showed that knock-down of UCA1 had inhibitory effects on glioma cell growth, cell invasion and migration, while overexpression of UCA1 promoted cell proliferation, cell invasion and migration in glioma cells. Collectively, these results suggested the oncogenic role of UCA1 in the glioma progression.

Wnt/β-catenin signaling controls myriad biological processes throughout development and adult life of many kinds of animals [34]. Aberrant Wnt/β-catenin signaling underlies a wide range of pathologies, especially cancer in humans [34]. In glioma cancer development, Wnt/β-catenin signaling was found to be an important signaling pathway. Activation of Wnt/β-catenin/Tcf signaling pathway was found in human glioma and interruption of β-catenin suppresses the epidermal growth factor receptor pathway by blocking multiple oncogenic targets in human glioma cells [35, 36]. Clinically, the protein levels of Wnt1, β-catenin and cyclin D1 were positively correlated with the Karnofsky performance scale score and World Health Organization grades of patients with glioma [19]. More importantly, Wnt/β-catenin was also found to interact with the lncRNA, CCND2 Antisense RNA 1 to regulate glioma cell proliferation [37]. In our study, we demonstrated that knock-down of UCA1 suppressed the activities of Wnt/β-catenin signaling in the glioma cells, and more importantly overexpression of UCA1 also increased the activities of Wnt/β-catenin signaling in the tumor tissues isolated from the nude mice. These results may indicate that UCA1 exerted its oncogenic function via modulating the activity of Wnt/β-catenin signaling in glioma.

The UCA1 was found to be a predictor for poor prognosis in cancer development. A meta-analysis from Wang et al., showed that UCA1 was a common molecular marker for lymph node metastasis and prognosis in various types of cancers [38]. In our study, the up-regulation of UCA1 was correlated with poor survival in patients with glioma. Our *in vitro* results also showed that UCA1 promoted chemo-resistance, which was consistent with studies in bladder cancer [39], and the rescue experiments showed that the effects of UCA1 on the chemoresistance of glioma may be associated with the modulation of Wnt/β-catenin signaling. However, in the clinical follow-up study, we have not examined whether UCA1 expression in glioma tissues was correlated with the chemo-sensitivity in patients with glioma, and further studies have to be conducted to address this concern.

In the present study, there are several limitations. The present study employed the xenograft nude mice model to determine the *in vivo* effects of UCA1, and further study may use the orthotopic glioma models to further confirm our findings. The monolayer system was used throughout the in vitro studies, and the effects of UCA1 on the spheroidic glioma cell progression may be a future direction of our study. The classification of glioma in the present study has not determined the isocitrate dehydrogenase mutation status, which was a limitation of the present study. The present study only determined the interaction between UCA1 and Wnt/β-catenin signaling pathway, and future studies may explore if UCA1 also affect other signaling pathways to regulate glioma progression.

## CONCLUSION

In conclusion, our results showed that UCA1 had a functional role in the regulation of glioma cell growth, invasion and migration, and chemo-resistance possibly via Wnt/β-catenin signaling pathway. More importantly, high expression of UCA1 predicted poor clinical outcome in glioma patients. UCA1 may be a potential therapeutic target for glioma treatment, though further studies are required to thoroughly understand the mechanisms of UCA1 in glioma development.

## MATERIALS AND METHODS

### Cell lines and culture conditions

The human astrocytes were purchased from Thermo Fisher Scientific (Waltham, USA) and were cultured in the GIBCO Astrocyte Medium (Thermo Fisher Scientific). The glioma cell lines including SHG44, U251, U87 and SHG139 were purchased from the ATCC (Manassas, USA), and the cells were cultured in the DMEM medium with 10% fetal bovine serum (FBS; Thermo Fisher Scientific). Cells were kept in the incubator supplied with 5% carbon dioxide at 37 °C. Human astrocyte cells were used as a normal control.

### SiRNAs, plasmids constructs, compounds and transfection

The siRNAs for UCA1 knock-down i.e. UCA1(a) (sense: 5′-GAGCCGAUCAGACAAACAAUU-3′; antisense: 5′-UUGUUUGUCUGAUCG GCUCUU-3′) and UCA1(b) (sense: 5′-CCUGGAAGCCACAAGAUUATT-3′; antisense: 5′-UAAUCUUGUGGCUUCCAGGAG-3′) as well as the scrambled control siRNA (sense: 5′-UUCGUCUGUACUCCACAUATT-3′; antisense: 5′- GAUGUCUUCUACAGUCCGATT-3′) were purchased from GenePharma (Shanghai, China). The empty plasmids, pcNDA3.1, and the plasmids overexpressing UCA1, i.e. pcDNA3.1-UCA1 were all synthesized by Shanghai BlueGene Biotech company (Shanghai, China). The compounds, lithium chloride (LiCl), XAV939 were purchased from Sigma (St. Louis, USA). The siRNAs and plasmids transfection for glioma cell lines were performed by using the Lipofectamine 2000 reagent (Invitrogen, Carlsbad, USA) according to the manufacturer’s instructions.

### Patients and glioma tissues collection

A total of 64 patients who were diagnosed with glioma were recruited in the study. All glioma tissues and adjacent normal brain tissues were collected from these patients when they underwent surgical resection in the First Teaching Hospital of Jilin University from January, 2014 to June, 2016. Inclusion criteria: (i) World Health Organization graded gliomas confirmed by histopathology; (ii) primary brain tumors; (iii) subjects had not undergone neoadjuvant therapy before surgery. The study was approved by the Ethics Committee of the First Teaching Hospital of Jilin University, and written consent were obtained from all the enrolled patients. The tissues were snap-frozen in the liquid nitrogen immediately after collection and kept in −80 °C until used.

### Quantitative real-time PCR (qRT-PCR)

The RNA from cell lines and tissues were extracted using TRIzol reagent (Invitrogen) according to the manufacturer’s protocol. The extracted RNA was reversely transcribed into cDNA by using the Primer Script RT reagent Kit (Takara, Dalian, China). Quantitative RT-PCR was performed using the Fast SYBR Green Master Mix (Applied Biosystems, Foster City, USA) and the ABI 7900 System (Life Technologies, New York, USA). Glyceraldehyde 3-phosphate dehydrogenase was used an internal control and the gene expression was calculated using 2^-ΔΔCt^ method.

### Western blotting assay

The proteins from cell lines were extracted using radioimmunoprecipitation assay buffer containing protease inhibitors (Roche, Basel, Switzerland). The extracted proteins were separated on the sodium dodecyl sulfate polyacrylamide gel electrophoresis gel. The separated proteins were transferred to polyvinylidene fluoride membrane, and the membranes were blocked with 5% skimmed milk at room temperature for 1 h. Then the membrane was incubated with different primary antibodies (Bcl-2, active caspase-3, active caspase-9, axin, non-phospho active β-catenin (Ser33/37/Thr41), cyclin D1, and β-actin antibodies) overnight at 4 °C. All the antibodies were purchased from the Abcam company (Cambridge, UK). After incubation with primary antibodies, the membranes were further incubated with horseradish peroxidase-conjugated secondary antibody. The Super-signal West Pico ECL Chemiluminescence kit (Thermo Scientific, Rockford, USA) was used to visualize the protein expression bands.

### CCK-8 assay

According to the manufacturer’s instructions, the CCK-8 solution was added into each well at 0, 24, 48 and 72 h after the transfection of UCA1 siRNAs, scrambled siRNA, pcDNA3.1 or pcDNA3.1-UCA1, and then the cells were incubated at 37 °C in a 5% CO_2_ incubator for 2 h. The OD value was measured at 450 nm using a microplate reader (Bio-Tek, Winooski, USA) with a reference wavelength of 630 nm.

### Cell apoptosis assay

At 48 h after transfection with UCA1 siRNAs or scrambled siRNA in glioma cells, the transfected cells were washed with phosphate buffered saline, and then the cells were incubated with Annexin V-FITC and propidium iodide dye at room temperature in the dark for 15 min. The cell apoptosis was detected using flow cytometry (BD Biosciences, Bedford, USA). The cell apoptosis was analyzed by using the CellQuest software (BD Biosciences).

### Cell invasion assay

Transwell invasion assay were performed using Matrigel Invasion Chambers (BD Biosciences) with inserts containing an 8-μm pore-sized membrane with a thin layer of Matrigel. At 48 h after transfection with UCA1 siRNAs or scrambled siRNA, or co-treatment with UCA1 siRNA and LiCl, pcDNA3.1, or pcDNA3.1-UCA1, the transfected cells were seeded on the upper chamber with serum-free medium. For the lower chamber, 0.5 ml medium supplemented with 10% FBS was added, and the transwell-containing plates were incubated for 8 h. After incubation, cells entering the lower surface of the filter chamber were fixed with 70% ethanol and stained with 0.1% crystal violet for 15 min at room temperature. The cells remaining on the upper surface of the filter membrane were removed by a cotton swab. The number of invaded cells was counted under a light microscope.

### Wound healing assay

At 48 h after transfection with UCA1 siRNAs or scrambled siRNA, or co-treatment with UCA1 siRNA and LiCl, pcDNA3.1, or pcDNA3.1-UCA1, the cells were seeded onto a six-well plate. After cells had grown to confluence, a scratch was created in the confluent monolayer in each well using a 100 μl pipette tip and further cultured for 24 h. Cells were photographed at 0 and 24 h under a light microscope.

### Determination of IC50 of cisplatin and temozolomide (TMZ) in glioma cells

At 48 h after glioma cells were treated with empty vector (pcDNA3.1), UCA1-overexpressing vector (pcDNA3.1-UCA1), UCA1-overexpressing vector (pcDNA3.1-UCA1) + XAV939, scrambled siRNA, siUCA1(a), or siUCA1(a) + LiCl; the treated cells were further treated with different concentrations of cisplatin (0 -300 μM) or TMZ (0 – 300 μM) for 48 h. The cell growth was analyzed by the CCK-8 assay as described above.

### TOP-FLASH assay

Glioma cells with TOP-FLASH reporter constructs and Renilla luciferase vector transfection were treated with UCA1 siRNAs, scrambled siRNA, LiCl or the vehicle negative control. At 48 h post-treatment, the TOP-FLASH activity was measured using the Dual-Luciferase Reporter Assay system (Promega, Madison, USA) according to the manufacturer’s protocol.

### In vivo tumor growth assay

Male BALB/c nude mice were obtained from Shanghai SLRC Laboratory Animal Co., Ltd (Shanghai, China). A U87 cell xenograft model was set up in the nude mice by subcutaneously injecting U87 cells with UCA1 overexpression or control U87 cells (5 x 10^6^) in to the right flanks of the nude mice. The tumor volume was monitored every 5 days for up to 30 days. The tumor volumes were calculated on the bases of caliper measurements using the following formula: tumor volume = (length x width x width)/2. At 30 days after cell injection, the mice were killed by cervical dislocation and tumor tissues were isolated for further analysis. All animal experiments were approved by the Research Ethics Committee of the First Teaching Hospital of Jilin University.

### Statistical analysis

All the statistical analysis was performed by using the GraphPad Prism Version 5.0 software (GraphPad Software, La Jolla, USA). Significant differences between two groups were examined by Student’s t test. Significant differences in more than two groups were examined by one-way ANOVA. The significant differences between low expression and high expression of UCA1 groups in the Kaplan-Meier curve were examined by log-rank test. P values less than 0.05 were considered statistically significant.

## Supplementary Material

Supplementary Figure
