# Supplementary material for: The long non-coding RNA, urothelial carcinoma associated 1, promotes cell growth, invasion, migration, and chemo-resistance in glioma through Wnt/β-catenin signaling pathway
Source: Aging (Albany NY). 2019 Oct 8;11(19):8239–53. doi: 10.18632/aging.102317 (PMC6814589; doi:10.18632/aging.102317)
Supplement: Supplementary Figure [file aging-11-102317-s001.pdf]

SUPPLEMENTARY FIGURE

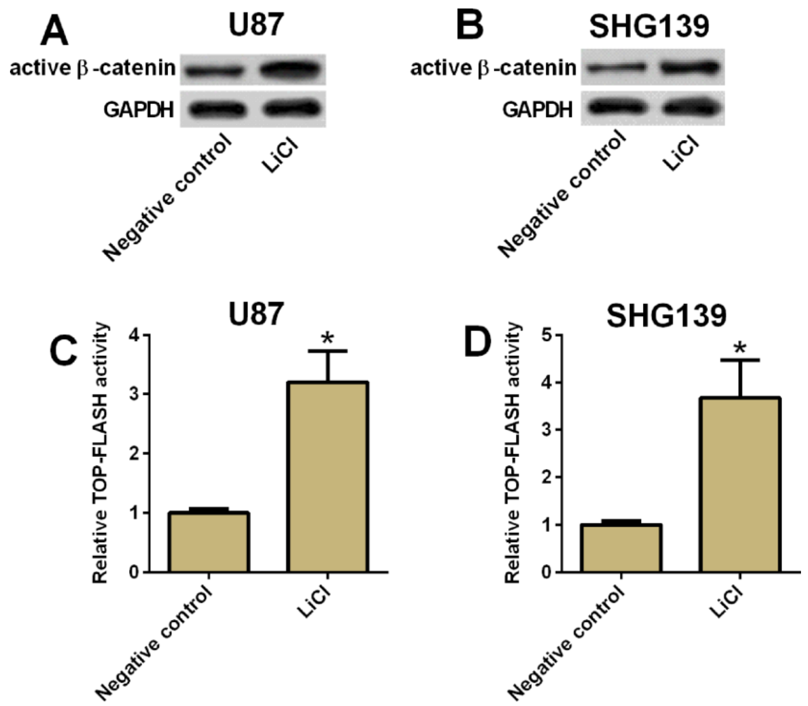

Supplementary Figure 1. LiCl treatment activated Wnt/β-cateinin signaling in U87 and SHG139 cells. The protein expression levels of active β-cateinin in (**A**) U87 and (**B**) SHG139 cells after LiCl or vehicle treatment were determined by western blotting assay. The TOP-FLASH activity in (**C**) U87 and (**D**) SHG139 cells after LiCl or vehicle treatment were determined by TOP-FLASH assay. Significant differences compared to control group were expressed as *P<0.05.
